# Growth monitoring of greenhouse lettuce based on a convolutional neural network

**DOI:** 10.1038/s41438-020-00345-6

**Published:** 2020-08-01

**Authors:** Lingxian Zhang, Zanyu Xu, Dan Xu, Juncheng Ma, Yingyi Chen, Zetian Fu

**Affiliations:** 1 China Agricultural University, Beijing, 100083 China; 2 Key Laboratory of Agricultural Informationization Standardization, Ministry of Agriculture and Rural Affairs, Beijing, China; 3 Institute of Environment and Sustainable Development in Agriculture, Chinese Academy of Agricultural Sciences, Beijing, 100081 China

**Keywords:** Developmental biology, Plant breeding

## Abstract

Growth-related traits, such as aboveground biomass and leaf area, are critical indicators to characterize the growth of greenhouse lettuce. Currently, nondestructive methods for estimating growth-related traits are subject to limitations in that the methods are susceptible to noise and heavily rely on manually designed features. In this study, a method for monitoring the growth of greenhouse lettuce was proposed by using digital images and a convolutional neural network (CNN). Taking lettuce images as the input, a CNN model was trained to learn the relationship between images and the corresponding growth-related traits, i.e., leaf fresh weight (LFW), leaf dry weight (LDW), and leaf area (LA). To compare the results of the CNN model, widely adopted methods were also used. The results showed that the values estimated by CNN had good agreement with the actual measurements, with *R*^2^ values of 0.8938, 0.8910, and 0.9156 and normalized root mean square error (NRMSE) values of 26.00, 22.07, and 19.94%, outperforming the compared methods for all three growth-related traits. The obtained results showed that the CNN demonstrated superior estimation performance for the flat-type cultivars of Flandria and Tiberius compared with the curled-type cultivar of Locarno. Generalization tests were conducted by using images of Tiberius from another growing season. The results showed that the CNN was still capable of achieving accurate estimation of the growth-related traits, with *R*^2^ values of 0.9277, 0.9126, and 0.9251 and NRMSE values of 22.96, 37.29, and 27.60%. The results indicated that a CNN with digital images is a robust tool for the monitoring of the growth of greenhouse lettuce.

## Introduction

Growth monitoring is essential for optimizing management and maximizing the production of greenhouse lettuce. Leaf fresh weight (LFW), leaf dry weight (LDW), and leaf area (LA) are critical indicators for characterizing growth^1,2^. Monitoring the growth of greenhouse lettuce by accurately obtaining growth-related traits (LFW, LDW, and LA) is of great practical significance for improving the yield and quality of lettuce^3^. The traditional methods for measuring growth-related traits, which are relatively straightforward, can achieve relatively accurate results^4^. However, the methods require destructive sampling, thus making it time-consuming and laborious^5–7^.

In recent years, nondestructive monitoring approaches have become a hot research topic. With the development of computer vision technology, image-based approaches have been widely applied to the nondestructive monitoring of crop growth^6,8–10^. Specifically, the image-based approaches extract low-level features from digital images and establish the relationship between the low-level features and manually measured growth-related traits, such as LA, LFW, and LDW. Based on this relationship, the image-derived features can estimate the growth-related traits, thus achieving nondestructive growth monitoring. For example, Chen et al.^6^ proposed method for the estimation of barley biomass. The authors extracted structure properties, color-related features, near-infrared (NIR) signals, and fluorescence-based features from images. Based on the above features, they built multiple models, i.e., support vector regression (SVR), random forest (RF), multivariate linear regression (MLR), and multivariate adaptive regression splines, to estimate barley biomass. The results showed that the RF model was able to accurately estimate the biomass of barley and better quantify the relationship between image-based features and barley biomass than the other methods. Tackenberg et al.^11^ proposed a method for estimating the growth-related traits of grass based on digital image analysis. Image features, such as the projected area (PA) and proportion of greenish pixels, were extracted, which were then fitted to the actual measured values of the aboveground fresh biomass, oven-dried biomass, and dry matter content by linear regression (LR). The results showed that all the determined coefficients of the constructed models were higher than 0.85, indicating that these features exhibited good linear relationship with growth-related traits. Casadesús and Villegas^5^ used color-based image features to estimate the leaf area index (LAI), green area index (GAI), and crop dry weight biomass (CDW) of two genotypes of barley. The image features included the H component of the HSI color space, the a* component of the CIEL*a*b* color space, and the U components of the CIELUV color space. In addition, the green fraction and greener fraction were also extracted. The features were linearly fitted to the measured values of LAI, GAI, and CDW at different growth stages. The results showed that the image features based on color had strong correlations with growth-related traits. Fan et al.^12^ developed a simple visible and NIR (near-infrared) camera system to capture time-series images of Italian ryegrass. Based on the digital number values of the R, G, and NIR channels of the raw images, MLR models for LAI estimation were built. The results showed that the image features derived from segmented images yielded better accuracy than those from non-segmented images, with an *R*^2^ value of 0.79 for LAI estimation. Liu and Pattey^13^ extracted the vertical gap fraction from digital images captured from nadir to estimate the LAI of corn, soybean, and wheat. Prior to the extraction of the canopy vertical gap fraction, the authors adopted the histogram-based threshold method to segment the green vegetative pixels. The results showed that the LAI estimated by the digital images before canopy closure was correlated with the field measurements. Sakamoto et al.^14^ used vegetation indices derived from digital images, i.e., the visible atmospherically resistant index (VARI) and excess green (ExG), to estimate the biophysical characteristics of maize during the daytime. The results showed that the VARI could accurately estimate the green LAI, and the ExG was able to accurately estimate the total LAI.

Although computer vision-based methods for estimating growth-related traits have achieved promising results, they are subject to two issues. First, the methods are susceptible to noise. Since the images are captured under field conditions, noise caused by uneven illumination and cluttered backgrounds is inevitable, which will affect image segmentation and feature extraction, thus potentially reducing the accuracy^15^. Second, the methods greatly rely on manually designed image features, which have large computational complexity. Moreover, the generalization ability of the extracted low-level image features is poor^16,17^. Therefore, a more feasible and robust approach should be explored.

Convolutional neural networks (CNNs), which is a state-of-the-art deep learning approach, can directly take images as input to automatically learn complex feature representations^18,19^. With a sufficient amount of data, CNNs can achieve better precision than conventional methods^20,21^. Therefore, CNNs have been used in a wide range of agricultural applications, such as weed and crop recognition^19,22,23^, plant disease diagnosis^24–28^, and plant organ detection and counting^21,29^. However, despite its extensive use in classification tasks, CNNs have rarely been applied to regression applications, and there are few reports on how CNNs have been used for the estimation of growth-related traits of greenhouse lettuce. Inspired by Ma et al.^18^, who accurately estimated the aboveground biomass of winter wheat at early growth stages by using a deep CNN, which is a CNN with a deep network structure, this study intended to adopt a CNN to construct an estimation model for growth monitoring of greenhouse lettuce based on digital images and to compare the results with conventional methods that have been widely adopted to estimate growth-related traits.

The objective of this study is to achieve accurate estimations of growth-related traits for greenhouse lettuce. A CNN is used to model the relationship between an RGB image of greenhouse lettuce and the corresponding growth-related traits (LFW, LDW, and LA). By following the proposed framework, including lettuce image preprocessing, image augmentation, and CNN construction, this study will investigate the potential of using CNNs with digital images to estimate the growth-related traits of greenhouse lettuce throughout the entire growing season, thus exploring a feasible and robust approach for growth monitoring.

## Material and methods

### Greenhouse lettuce image collection and preprocessing

The experiment was conducted at the experimental greenhouse of the Institute of Environment and Sustainable Development in Agriculture, Chinese Academy of Agricultural Sciences, Beijing, China (N39°57′, E116°19′). Three cultivars of greenhouse lettuce, i.e., Flandria, Tiberius, and Locarno, were grown under controlled climate conditions with 29/24 °C day/night temperatures and an average relative humidity of 58%. During the experiment, natural light was used for illumination, and a nutrient solution was circulated twice a day. The experiment was performed from April 22, 2019, to June 1, 2019. Six shelves were adopted in the experiment. Each shelf had a size of 3.48 × 0.6 m, and each lettuce cultivar occupied two shelves.

The number of plants for each lettuce cultivar was 96, which were sequentially labeled. Image collection was performed using a low-cost Kinect 2.0 depth sensor^30^. During the image collection, the sensor was mounted on a tripod at a distance of 78 cm to the ground and was oriented vertically downwards over the lettuce canopy to capture digital images and depth images. The original pixel resolutions of the digital images and depth images were 1920 × 1080 and 512 × 424, respectively. The digital images were stored in JPG format, while the depth images were stored in PNG format. The image collection was performed seven times 1 week after transplanting between 9:00 a.m. and 12:00 a.m. Finally, two image datasets were constructed, i.e., a digital image dataset containing 286 digital images and a depth image dataset containing 286 depth images. The number of digital images for Flandria, Tiberius, and Locarno was 96, 94 (two plants did not survive), and 96, respectively, and the number of depth images for the three cultivars was the same.

**Fig. 1:**
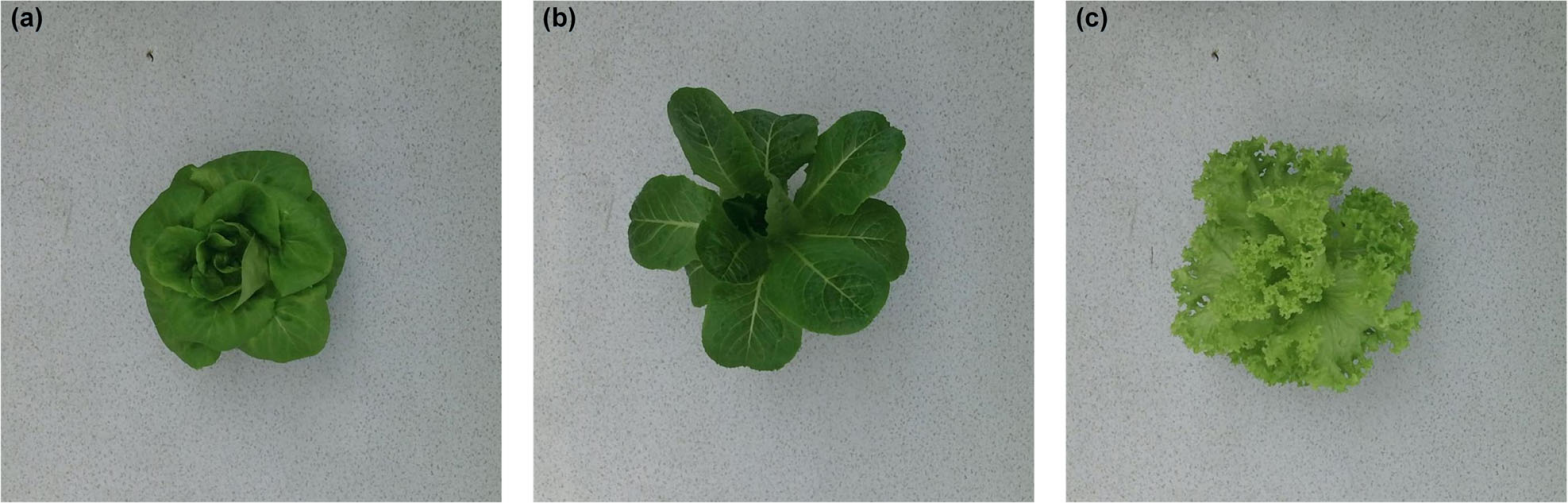
Examples of the digital images for the three cultivars. **a**, **b**, and **c** shows the cultivar of Flandria, Tiberius, and Locarno

**Fig. 2:**
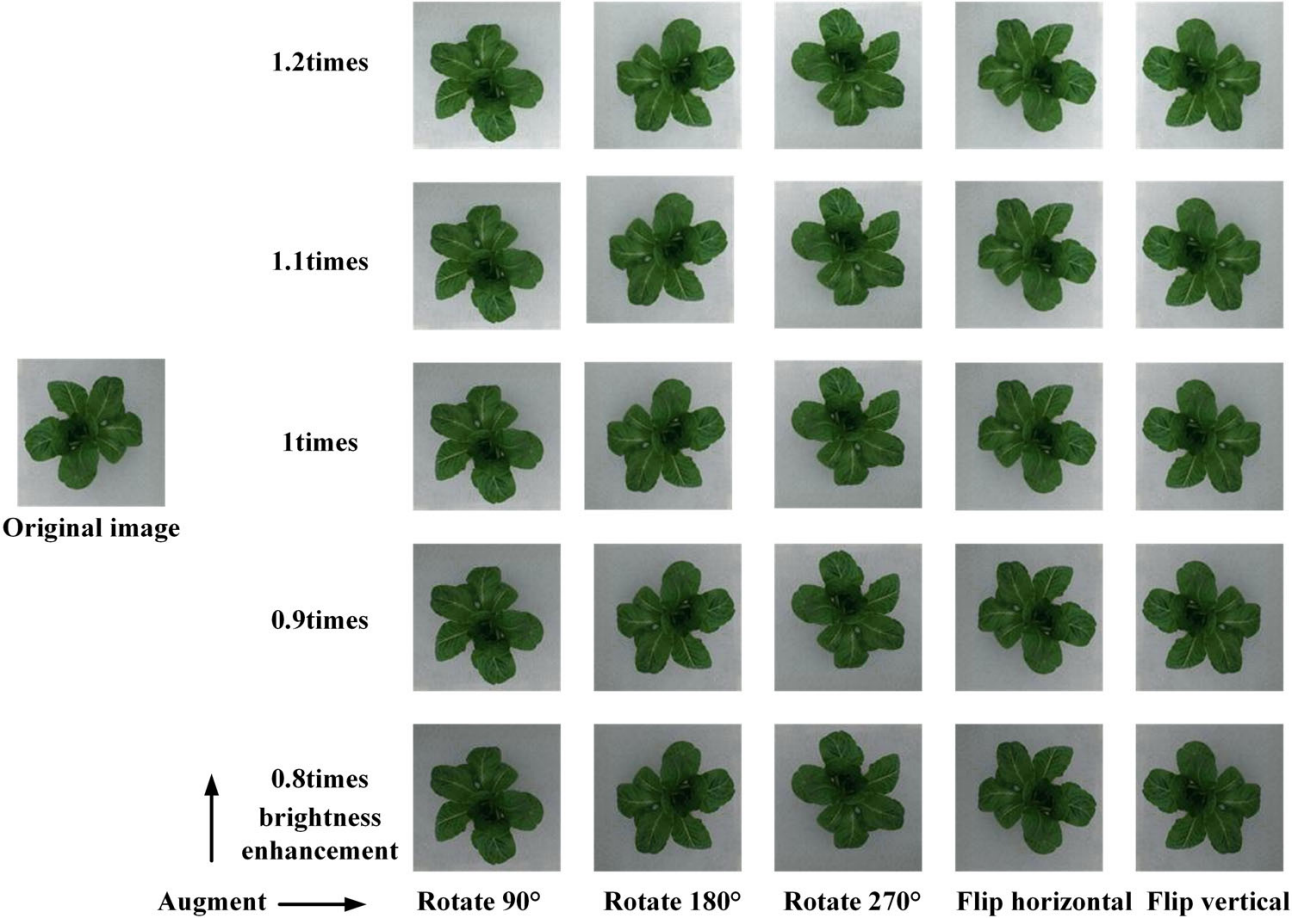
Image augmentation scheme

Since the original digital images of greenhouse lettuce contained an excess of background pixels, this study manually cropped images to eliminate the extra background pixels, after which images were uniformly adjusted to 900 × 900 pixel resolution. Figure 1 shows examples of the cropped digital images for the three cultivars. Prior to the construction of the CNN model, the original digital image dataset was divided into two datasets in a ratio of 8:2, i.e., a training dataset and a test dataset. The two datasets both covered all three cultivars and sampling intervals. The number of images for the training dataset was 229, where 20% of the images were randomly selected for the validation dataset. The test dataset contained 57 digital images. To enhance data diversity and prevent overfitting, a data augmentation method was used to enlarge the training dataset (Fig. 2). The augmentations were as follows: first, the images were rotated by 90°, 180°, and 270°, and then flipped horizontally and vertically. To adapt the CNN model to the changing illumination of the greenhouse, the images in the training dataset were converted to the HSV color space, and the brightness of the images was adjusted by changing the V channel^31^. The brightness of the images was adjusted to 0.8, 0.9, 1.1, and 1.2 times that of the original images to simulate the change in daylight. In total, the training dataset was enlarged by 26 times, resulting in 5954 digital images.

### Measurement of greenhouse lettuce growth-related traits

Field measurements of LFW, LDW, and LA were performed simultaneously with image collection. These measurements were conducted at an interval of seven days, specifically on April 29, May 6, May 13, May 20, May 27, May 31, and June 1 of 2019. For the first six measurements, ten plants of greenhouse lettuce were randomly sampled each time for each cultivar. The measurements were obtained using a destructive sampling method. The sample was placed on a balance with a precision of 0.01 g after root removal, and the LFW was measured. The LA of the corresponding sample was obtained by a LA meter (LI-3100 AREA METER; LI-COR Inc. Lincoln, Nebraska, USA). Given the relatively large leaves of lettuce during the late growing season, the sample was sealed in an envelope and oven-dried at 80 °C for 72 h, after which the sample was weighed to obtain the LDW. For the last measurement, all the remaining lettuce plants were harvested, and the measurements were obtained by using the same method.

### Construction of the CNN

**Fig. 3:**
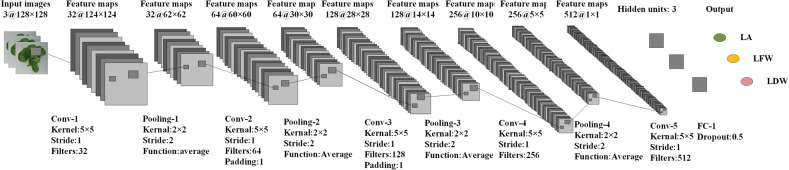
Architecture of the CNN model

The architecture of the CNN model is shown in Fig. 3. The CNN model consisted of five convolutional layers, four pooling layers, and one fully connected layer. The input to the CNN model was digital images of greenhouse lettuce with a size of 128 × 128 × 3 (width × height (H) × channel). The convolutional layers adopted kernels with a size of 5 × 5 to extract features. The number of kernels in the five convolutional layers were 32, 64, 128, 216, and 512. To keep the size of the feature maps as an integer, zero-padding was employed in the second and third convolutional layers. The kernels in the pooling layers had a size of 2 × 2 and a stride of 2, which was able to reduce the size of feature maps by a factor of two. The average pooling function was adopted in the pooling layers instead of the max pooling function. The number of hidden neurons in the fully connected layer was three, corresponding to the three outputs of the model, i.e., the LFW, LDW, and LA. Therefore, the CNN model could estimate the three growth-related traits simultaneously. Dropout was used, and the rate was 0.5. In this study, the CNN model used stochastic gradient descent to optimize the network weights. The initial learning rate of the model was set to 0.001 and dropped every 20 epochs by a drop factor of 0.1. The mini-batch size was set to 128, and the maximum number of epochs for training was set to 300.

### Performance evaluation

**Fig. 4:**
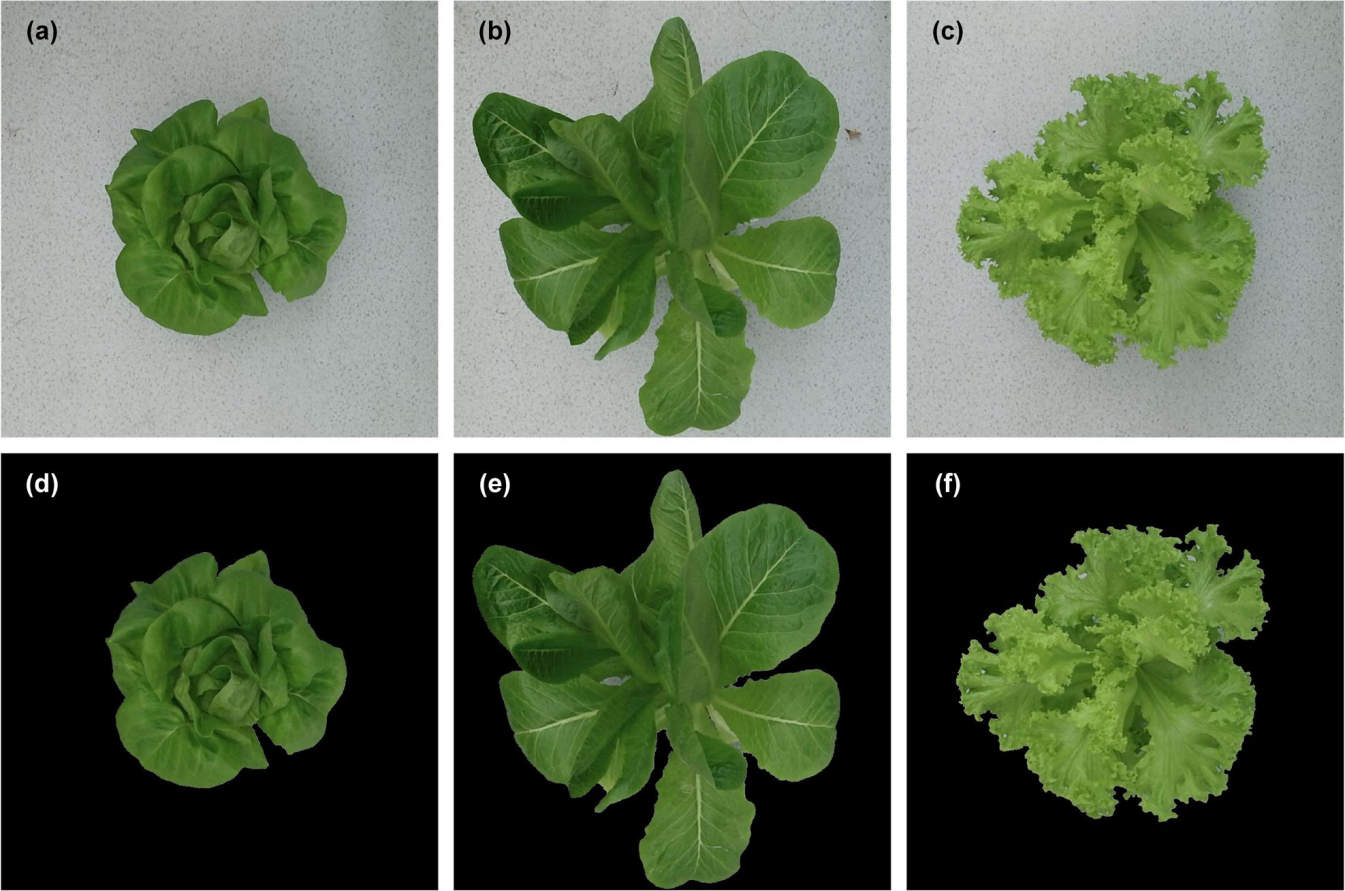
Image segmentation results of the three cultivars of greenhouse lettuce. **a**–**c** shows the original images of greenhouse lettuce, and **d**–**f** shows the corresponding segmentation results

To evaluate the performance of the CNN model, tests were performed with the widely adopted estimation methods. Two shallow machine learning classifiers, i.e., SVR^32,33^ and RF^34^, were adopted to estimate the growth-related traits of greenhouse lettuce since these two methods have been reported to achieve good performance in crop growth monitoring. According to “Greenhouse lettuce image collection and preprocessing,” there was a large number of background pixels in the captured images of greenhouse lettuce. Therefore, it was necessary to conduct image segmentation to extract the lettuce pixels, thus ensuring that the extracted features in the following step were presenting the lettuce plants. For the digital images of the greenhouse lettuce, since the color contrast between the lettuce plant and the background was very obvious, image segmentation was achieved by using the adaptive threshold method for the color information. Some segmentation results are shown in Fig. 4.

To build the shallow machine learning classifiers, feature extraction was performed on the segmented images of greenhouse lettuce. According to the characteristics of the three cultivars of greenhouse lettuce, low-level image features, including color, texture, and shape features, were extracted^35^. The color features included the average and standard deviation of 15 color components of five color spaces (RGB, HSV, CIEL*a*b, YCbCr, and HSI)^36^. Based on the color components, the gray level co-occurrence matrix^37^ was combined to extract the texture features. The texture features included the contrast, correlation, energy, and homogeneity of the 15 color components. The shape features of the greenhouse lettuce that were extracted were area and perimeter in this study. The area was the area enclosed by the outline, and the perimeter was the total length of the blade outline. After extracting the image features, the Pearson coefficient was used to perform correlation analysis between the extracted features and the actual values of the LFW, LDW, and LA of greenhouse lettuce. The features with relatively high correlation values were used to build the shallow machine learning classifiers.

**Fig. 5:**
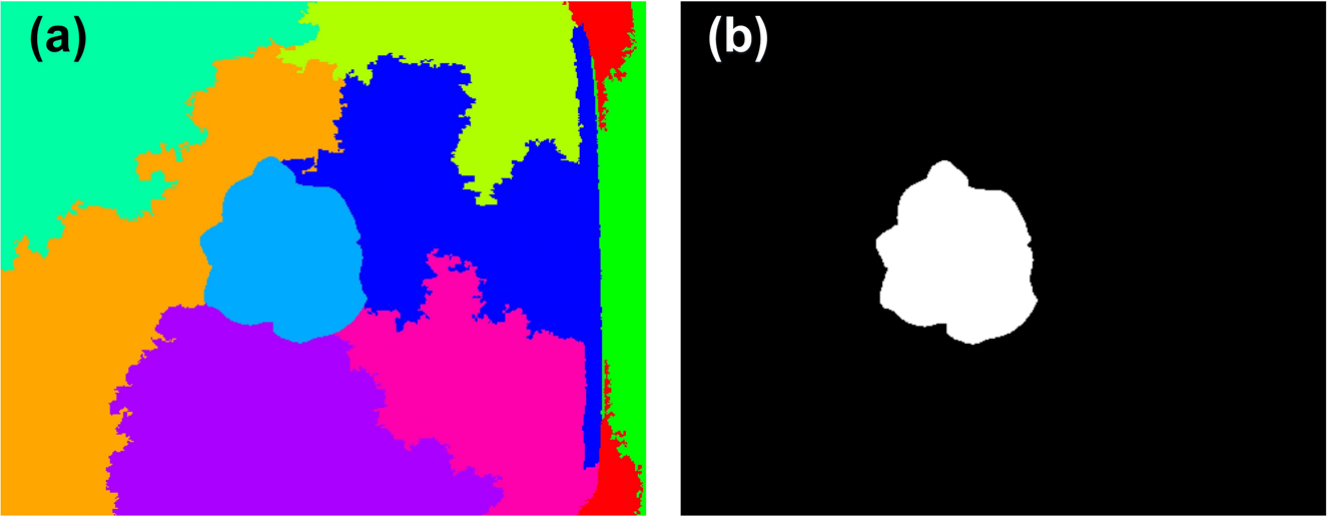
Depth image segmentation. **a** shows the randomly colored superpixels, and **b** show the segmented lettuce plant

**Fig. 6:**
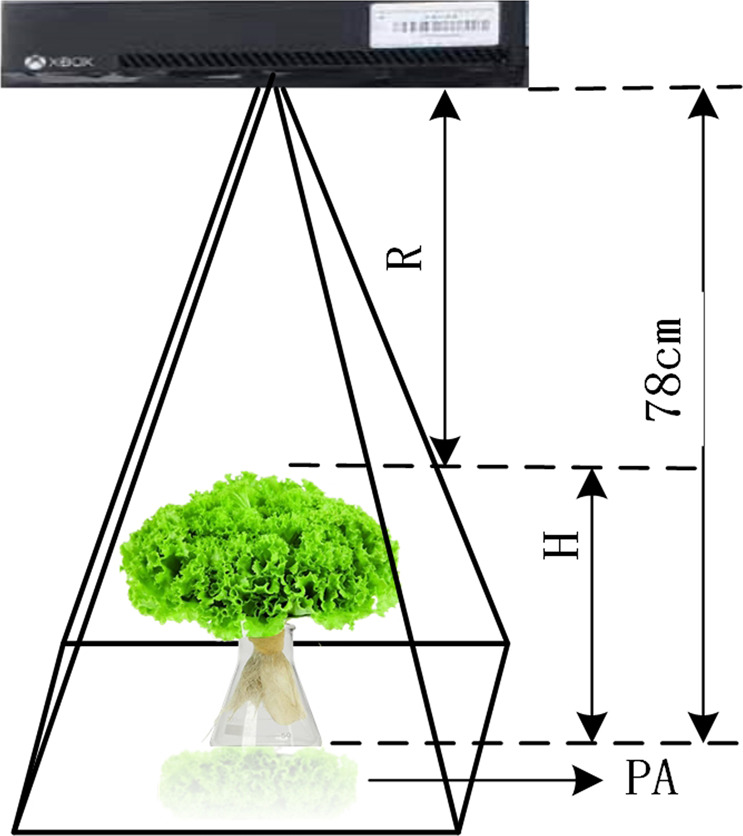
The calculations for PA and H

In addition to the above image features, structural features derived from the depth images, including H, PA, and digital volume (V), were also used to estimate the growth-related traits of the greenhouse lettuce^8,38–40^. Three LR models using H, PA, and V as the predictor variables (LR-H, LR-PA, and LR-V) were also used for comparison. Similar to the processing of digital images, image segmentation was also conducted on the depth images, which was achieved by the entropy rate superpixel segmentation method^41^. The lettuce plant could be extracted using the Euclidean distance to find the superpixel that was closest to the center of the image (Fig. 5). Once the lettuce plant was obtained, the structural features could be calculated (Fig. 6). Since the pixel value of the depth image was the actual distance from the sensor to the object, it reflected the depth information. Therefore, PA could be obtained by counting the number of pixels in the lettuce plant area. H could be obtained by averaging the H of the pixels in the lettuce plant area, which was obtained by using the H of the sensor minus the pixel values in the lettuce plant area. V could be obtained by multiplying PA by H.

In this study, the coefficient of determination (*R*^2^) and the normalized root mean square error (NRMSE) were used as the criteria for evaluating the performances of all the estimation models.

## Results

In this study, the construction of the estimation models and image preprocessing were implemented using MATLAB 2018b (MathWorks Inc., USA). The software environment was Windows 10 Professional Edition, the hardware environment was an Intel i7 processor, CPU 3.20 GHz, with 8 GB memory, and the GPU was NVIDIA GeForce GTX1060.

### Estimation results of the CNN model

**Fig. 7:**
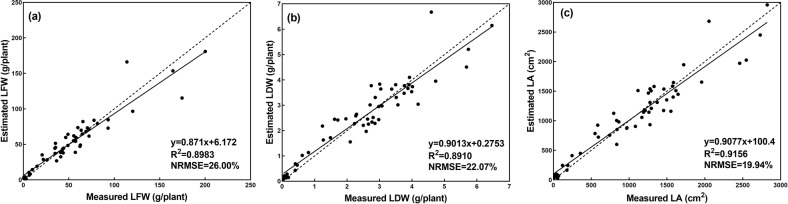
Estimation results of growth-related traits based on the CNN model. **a**, **b**, and **c** shows the estimation results of LFW, LDW, and LA, dashed line indicates the 1:1 line

**Fig. 8:**
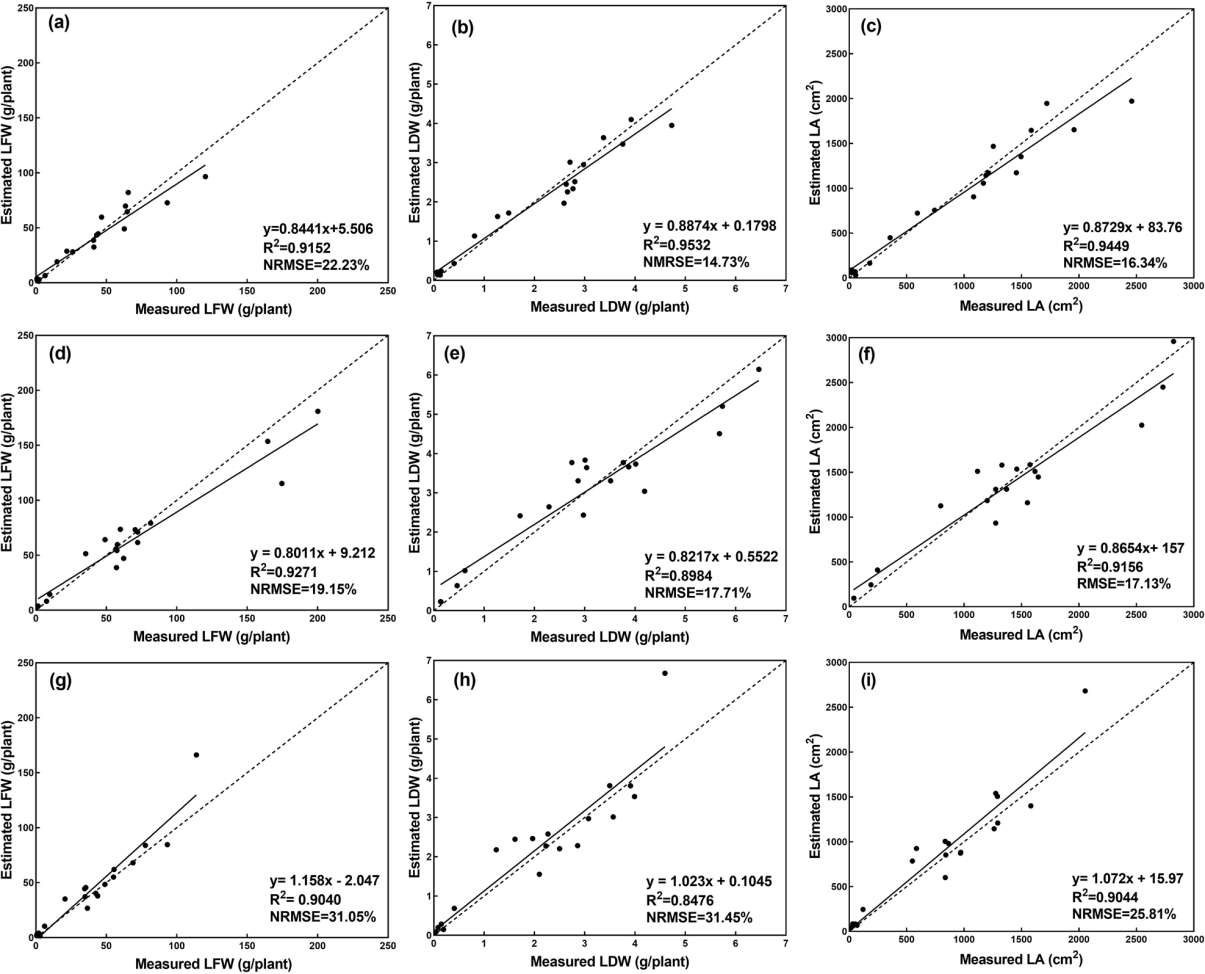
Estimation results of the growth-related traits for each greenhouse lettuce cultivar. **a**–**c** shows the estimation results of LFW, LDW, and LA for Flandria, **d**–**f** shows the estimation results of LFW, LDW, and LA for Tiberius, **g**–**i** shows the estimation results of LFW, LDW, and LA for Locarno, the dashed line indicates the 1:1 line

When the training process finished, the test dataset was used to test the performance of the CNN model. The performance of the CNN model evaluated over the test dataset is shown in Fig. 7. The results showed strong correlations between the actual measurements of the growth-related traits and those estimated by the CNN model. It can also be seen that the CNN model demonstrated the best performance on the estimation of LA, achieving the highest *R*^2^ and the lowest NRMSE (*R*^2^ = 0.9156, NRMSE = 19.94%). The performance of the CNN model for LFW and LDW was similar, with *R*^2^ values of 0.8983 and 0.8910, respectively, and NRMSE values of 26.00% and 22.07%, respectively. For the lettuce cultivars (Fig. 8 and Table 1), the CNN model showed different performances. Generally, the CNN model was better at estimating the growth-related traits of Flandria and Tiberius than Locarno, which might be due to the differences in the leaf shape of the lettuce. Flandria and Tiberius have flat-leaf types with relatively stretched leaves, while Locarno is a curled-leaf type with uneven curling leaves. Therefore, the CNN model was able to obtain more comprehensive information when extracting the features of Flandria and Tiberius. However, the leaves of Locarno were more curled, resulting in the learned features not being comprehensive enough to account for the covering and hiding of leaf sections, which affected the estimation accuracy. Therefore, the CNN model achieved the highest prediction accuracy for Flandria and Tiberius.

**Table 1:** Results of the estimated growth-related traits for the three cultivars of greenhouse lettuce by the CNN model

Cultivars	LFW		LDW		LA	
	*R*^2^	NRMSE	*R*^2^	NRMSE	*R*^2^	NRMSE
Flandria	0.9152	22.23%	0.9532	14.73%	0.9449	16.34%
Tiberius	0.9271	19.15%	0.8984	17.71%	0.9156	17.13%
Locarno	0.9040	31.05%	0.8476	31.45%	0.9044	25.81%

### Comparison of the results with the conventional estimation methods

Prior to the construction of shallow machine learning classifiers, correlation analysis was performed between pairs of parameters that included the low-level image features and the three growth-related traits. The features that were highly correlated to the actual values of the growth-related traits were used to build the classifiers. The selected features for building the classifiers to estimate LFW, LDW, and LA are shown in Tables 2–4, respectively.

**Table 2:** Correlations of the LFW measurements with the low-level image features

Features	Correlation coefficient	Features	Correlation coefficient
RGB_G_Energy	−0.692^a^	YCrCb_Cr_Energy	−0.687^a^
HSV_H_Average	0.685^a^	YCrCb_Cb_Average	−0.700^a^
HSV_H_Energy	−0.687^a^	YCrCb_Cb_Energy	−0.687^a^
HSV_S_Average	0.691^a^	HSI_S_Average	−0.690^a^
LAB_L_Energy	−0.693^a^	HSI_S_Energy	−0.697^a^
LAB_A_Average	−0.696^a^	HSI_S_Homogeneity	−0.686^a^
LAB_B_Energy	−0.686^a^	Area	0.9187^a^

^a^Correlation is significant at the 0.01 level

**Table 3:** Correlations of the LDW measurements with the low-level image features

Features	Correlation coefficient	Features	Correlation coefficient
RGB_R_Homogeneity	−0.652^a^	LAB_B_Energy	−0.652^a^
RGB_G_Energy	−0.651^a^	YCrCb_Cr_Energy	−0.651^a^
HSV_H_Energy	−0.653^a^	YCrCb_Cb_Energy	−0.652^a^
HSV_S_Energy	−0.650^a^	HSI_S_Std	0.661^a^
HSV_S_Homogeneity	−0.657^a^	HSI_S_Energy	−0.653^a^
LAB_L_Energy	−0.655^a^	Area	0.8831^a^
HSI_S_Homogeneity	−0.661^a^		

^a^Correlation is significant at the 0.01 level

**Table 4:** Correlations of the LA measurements with the low-level image features

Features	Correlation coefficient	Features	Correlation coefficient
RGB_G_Energy	−0.664^a^	YCrCb_Cr_Energy	−0.662^a^
HSV_H_Average	0.660^a^	YCrCb_Cb_Average	−0.671^a^
HSV_H_Energy	−0.664^a^	YCrCb_Cb_Std	0.662^a^
HSV_S_Average	0.667^a^	YCrCb_Cb_Energy	−0.664^a^
HSV_S_Energy	−0.662^a^	HSI_S_Energy	−0.667^a^
LAB_L_Energy	−0.667^a^	HSI_S_Homogeneity	−0.665^a^
LAB_B_Energy	−0.663^a^	Area	0.8930^a^

^a^Correlation is significant at the 0.01 level

**Fig. 9:**
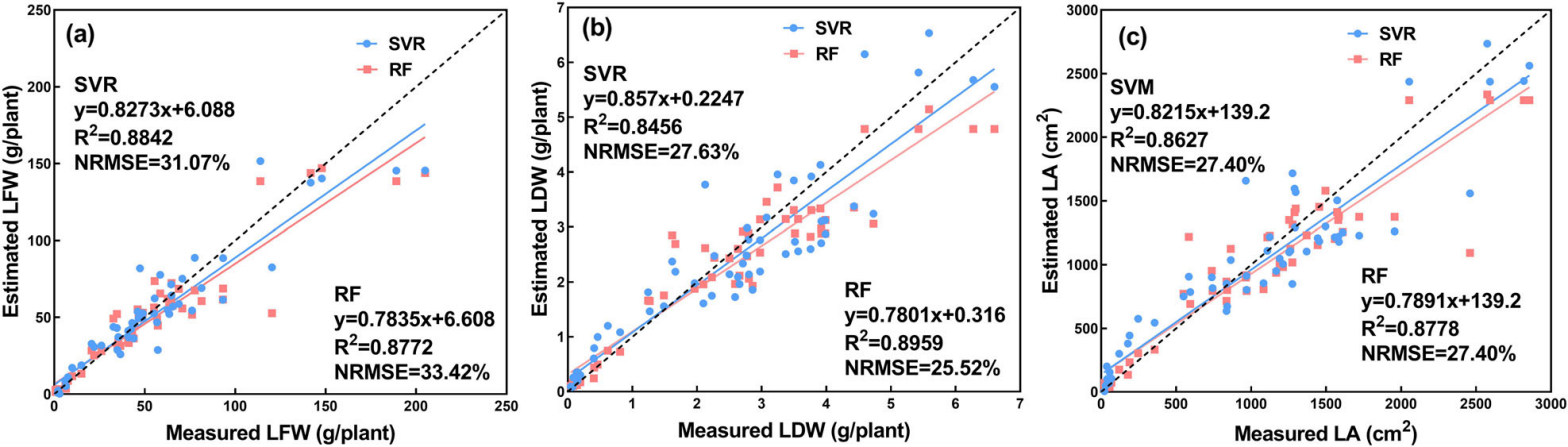
Estimation results of the growth-related traits based on SVR and RF. **a**, **b**, and **c** shows the estimation results of LFW, LDW, and LA, dashed line indicates the 1:1 line

Based on the selected features, SVR and RF models were constructed. The estimation results by the two classifiers are shown in Fig. 9. For the growth-related trait of LFW, SVR demonstrated better performance than RF, while for the growth-related traits of LDW and LA, RF achieved superior results to SVR. Compared with the performance of the CNN model (Table 5), although the *R*^2^ value of RF for estimating LDW was very close, its NRMSE value was approximately 3.5% higher. Considering the *R*^2^ and NRMSE comprehensively, the CNN model indicated better performance on estimating LDW than RF. According to Table 5, it can be concluded that the CNN models outperformed the two classifiers in estimating all three growth-related traits with higher *R*^2^ values and lower NRMSE values. A possible explanation for the results might be that the construction of SVR and RF was based on the low-level features of the digital images, which could be extracted based on the image segmentation of lettuce plants. This method may be unreliable due to an uneven external illumination and other factors, potentially resulting in a low accuracy image segmentation, which decreased the accuracy of the feature extraction^15^. Furthermore, the low-level image features were artificially designed, indicating that the generalization ability of SVR and RF models was poor^18,28^. Therefore, the estimation accuracy for the growth-related traits of the greenhouse lettuce was worse than that of the CNN model.

**Table 5:** Estimation results of the constructed models for the three growth-related traits

Models	LFW		LDW		LA	
	*R*^2^	NRMSE	*R*^2^	NRMSE	*R*^2^	NRMSE
CNN	0.8983	26.00%	0.8910	22.07%	0.9156	19.94%
SVM	0.8842	31.07%	0.8456	27.63%	0.8627	27.40%
RF	0.8772	33.42%	0.8959	25.52%	0.8778	27.40%
LR-V	0.8492	33.53%	0.7669	32.73%	0.7561	34.78%
LR-PA	0.8454	34.26%	0.8281	28.49%	0.8062	31.38%
LR-H	0.6193	53.85%	0.7074	36.83%	0.6846	39.84%

**Fig. 10:**
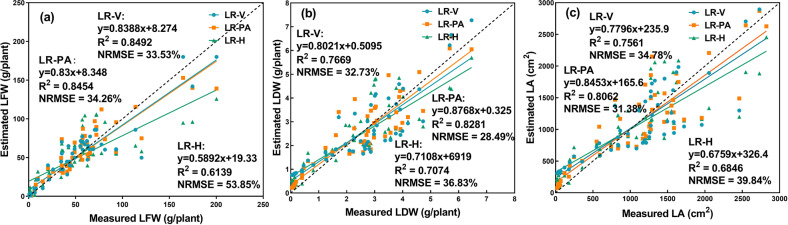
Estimation results of the growth-related traits based on the structural features. **a** LFW, **b** LDW, **c** LA, dashed line indicates the 1:1 line

It can be seen from Fig. 10 that the LR-V and LR-PA models demonstrated better estimation performance than the LR-H model. The research objects of this paper were vegetables. From the perspective of horticulture research, the H of lettuce was an essential trait for growth monitoring. However, it is not fair to say, the higher, the better. In the case of nutrient deficiencies, excessive growth can also occur^42^. Therefore, the estimated biomass (LFW and LDW) and LA from the H of greenhouse lettuce can be inaccurate. In addition, existing research has shown that V and PA have relatively strong correlations with the growth-related traits of crops^43,44^. Therefore, the results that the LR models that used V and PA as predictor variables exhibited better estimation accuracy than those using H as the predictor variable were within expectation. Compared with the CNN model (Table 5), the three LR models (LR-V, LR-PA, and LR-H) based on structural features had low *R*^2^ values and high NRMSE values. In addition, the two shallow machine learning classifiers outperformed the LR models (Table 5). This result might be explained by the fact that the growth of lettuce was not only related to geometric features but also related to the color and texture features of lettuce. The structural features derived from depth images were geometric, thus containing no color or texture information of the greenhouse lettuce. Therefore, the LR models based on structural features derived from depth images yielded the worst prediction accuracy.

### Generalization test results

The evaluation of the performance of the proposed estimation method using images from different growing seasons would strengthen the belief in its validity and generalization^45^. Therefore, we performed a generalization test by directly applying the pretrained CNN model to images of Tiberius planted in another growing season (Season 2). We adopted the same experimental design as in “Greenhouse lettuce image collection and preprocessing,” resulting in a dataset containing 200 images and corresponding growth-related traits covering the entire growing season of the greenhouse lettuce.

**Fig. 11:**
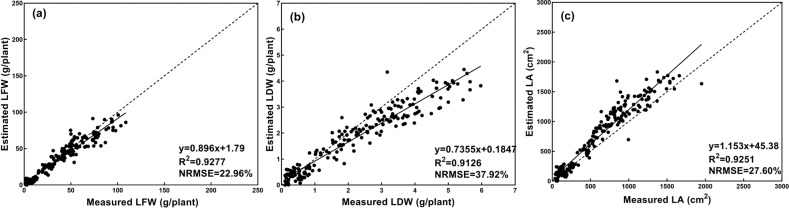
Estimation results of the generalization test. **a**, **b**, and **c** shows the estimation results of LFW, LDW, and LA, dashed line indicates the 1:1

The estimation results are shown in Fig. 11. Regression analysis suggested that the values of the three growth-related traits estimated from the images in Season 2 agreed well with the corresponding values derived from field measurements. For the three growth-related traits of LFW, LDW, and LA, the CNN model had *R*^2^ values equal to 0.9277, 0.9126, and 0.9251, respectively, and NRMSE values equal to 22.96%, 37.92%, and 27.60%, respectively. The results revealed that the proposed estimation method had a strong generalization ability. On the other hand, the temperature and humidity in the greenhouse changed with the seasons, which would cause the growth of lettuce to change. Promisingly, the estimation results of the CNN model were still accurate, demonstrating that the proposed estimation method achieved excellent robustness and made a reliable tool for monitoring the growth of greenhouse lettuce.

## Discussion

The close and accurate monitoring of crop growth is critical for the optimized management of crop production^46^. Direct measurement of growth-related traits is destructive and inefficient. Nondestructive monitoring has emerged and become a hot topic of current research. Computer vision technology has been widely used in nondestructive monitoring, providing great convenience to growth-related trait acquisition by using conventional methods, i.e., SVR, RF, and LR. However, these methods are limited in practical applications. Therefore, with the rapid development of deep learning, CNN has become preferred by researchers for its advantages, such as no need to manually extract features. In this study, we demonstrated that CNN can serve as a convenient and accurate tool to obtain growth-related traits for greenhouse lettuce. In comparison with the conventional methods, the proposed CNN model showed superior estimation performances in estimating the three growth-related traits for multiple cultivars of greenhouse lettuce, as shown in Table 5. Specifically, the estimated results of the CNN model on all three growth-related traits had *R*^2^ values above 0.89 and NRMSE values below 27%. The results demonstrated the advantages of the CNN model in that it was able to automatically learn complex feature representations from digital images, which can be translated to a strong generalization ability^47^. The obtained results agreed with previous studies by Ma et al.^18^ and Grinblat et al.^23^.

### Limitations and future work

Although the proposed method has been shown to be accurate and efficient, there are still limitations that we need to take into account. One limitation is that images were acquired from only the top view, indicating that the error may increase if there are too many overlaps between the leaves. Another limitation is the fixed H during image collection. If the H changes, the estimated results may be biased.

Future studies will continue to collect more images to enlarge our dataset, such as images of other lettuce cultivars. To improve the efficiency of the method, we will explore the growth-related traits of multiple lettuce plants in a single image. In addition, the factors that may influence the performance of the CNN model, such as stress and H for image collection, will also be explored.

### Prospective

Growth monitoring can indicate the status of greenhouse lettuce, which is critical for intelligent field management to control the greenhouse environment and establish nutrition strategies. The proposed estimation method allowed us to estimate LFW, LDW, and LA for multiple cultivars of greenhouse lettuce by using digital images, which are low cost and easy to use. The method has great potential for being used in the field when combined with mobile devices or when integrated into other automatic platforms since its input images can be captured by low-cost digital cameras.

## Conclusions

In this study, a method for the estimation of growth-related traits of multiple cultivars of greenhouse lettuce was proposed by using digital images and CNN, which could provide support for growth monitoring. The estimated growth-related traits had good agreement with the actual measurements, with *R*^2^ values of ~0.9 and NRMSE values of ~20%. Furthermore, the performance of the proposed method was superior to that of the conventional methods that are widely adopted to estimate growth-related traits. The obtained results showed that the proposed method in this study achieved better estimation performance for Flandria and Tiberius cultivars than Locarno. After another batch of images were acquired of the Tiberius cultivar that was planted in Season 2 for verification, the results reinforced that the proposed estimation method had a strong generalization ability, as well as robust estimation performance despite the seasonal factors. It can be concluded that the proposed method is a reliable tool for estimating the growth-related traits of greenhouse lettuce and has excellent potential in the application of growth monitoring. Furthermore, the accurate monitoring of growth-related traits can provide support for scientific management decision-making.

## Data Availability

The authors declare that all data supporting the findings of this study are available within the paper and its supplementary information files.

## Code availability

Computer program codes and image data used in this study can be accessed through https://figshare.com/s/4e27e3ba666d32daf5c5.

## References

[1] Teobaldelli M (2019). Developing an accurate and fast non-destructive single leaf area model for loquat (Eriobotrya japonica Lindl) cultivars. Plants.

[2] Lati RN, Filin S, Eizenberg H (2013). Estimation of plants’ growth parameters via image-based reconstruction of their three-dimensional shape. Agron. J..

[3] Bauer A (2019). Combining computer vision and deep learning to enable ultra-scale aerial phenotyping and precision agriculture: a case study of lettuce production. Hortic. Res..

[4] Levy PE, Jarvis PG (1999). Direct and indirect measurements of LAI in millet and fallow vegetation in HAPEX-Sahel. Agric. Meteorol..

[5] Casadesús J, Villegas D (2014). Conventional digital cameras as a tool for assessing leaf area index and biomass for cereal breeding. Plant Biol..

[6] Chen D (2018). Predicting plant biomass accumulation from image-derived parameters. Gigascience.

[7] Zhang L, Verma B, Stockwell D, Chowdhury S (2018). Density weighted connectivity of grass pixels in image frames for biomass estimation. Expert Syst. Appl..

[8] Hu Y, Wang L, Xiang L, Wu Q, Jiang H (2018). Automatic non-destructive growth measurement of leafy vegetables based on kinect. Sensors.

[9] Krishna G, Sahoo RN, Singh P, Bajpai V (2019). Comparison of various modelling approaches for water deficit stress monitoring in rice crop through hyperspectral remote sensing. Agric Water Manag..

[10] Yu J, Li C, Paterson AH (2016). High-throughput phenotyping of cotton plant height using depth images under field conditions. Comput. Electron. Agric.

[11] Tackenberg O (2007). A new method for non-destructive measurement of biomass, growth rates, vertical biomass distribution and dry matter content based on digital image analysis. Ann. Bot..

[12] Fan X (2018). A simple visible and near-infrared (V-NIR) camera system for monitoring the leaf area index and growth stage of Italian ryegrass. Comput. Electron. Agric..

[13] Liu J, Pattey E (2010). Retrieval of leaf area index from top-of-canopy digital photography over agricultural crops. Agric. Meteorol..

[14] Sakamoto T (2012). Application of day and night digital photographs for estimating maize biophysical characteristics. Precis. Agric..

[15] Ma J (2017). A segmentation method for greenhouse vegetable foliar disease spots images using color information and region growing. Comput. Electron. Agric..

[16] Wan, J., Wang, D., Hoi, S.C.H. & Wu, P. Deep learning for content-based image retrieval: a comprehensive study. In *Proc. 22nd ACM International Conference on Multimedia*, 157–166 (Istanbul, Turkey, 2014).

[17] Pound MP (2017). Deep machine learning provides state-of-the-art performance in image-based plant phenotyping. Gigascience.

[18] Ma J (2019). Estimating above ground biomass of winter wheat at early growth stages using digital images and deep convolutional neural network. Eur. J. Agron..

[19] Ferreira S, Freitas DM, Gonçalves G, Pistori H, Theophilo M (2017). Weed detection in soybean crops using ConvNets. Comput. Electron. Agric.

[20] Ghosal S (2018). An explainable deep machine vision framework for plant stress phenotyping. Proc. Natl Acad. Sci..

[21] Uzal LC (2018). Seed-per-pod estimation for plant breeding using deep learning. Comput. Electron. Agric..

[22] Dyrmann M, Karstoft H, Midtiby HS (2016). Plant species classification using deep convolutional neural network. Biosyst. Eng..

[23] Grinblat GL, Uzal LC, Larese MG, Granitto PM (2016). Deep learning for plant identification using vein morphological patterns. Comput. Electron. Agric..

[24] Nachtigall, L. G., Araujo, R. M. & Nachtigall, G. R. Classification of apple tree disorders using convolutional neural networks. In *Proc. 2016 IEEE 28th International Conference on Tools with Artificial Intelligence* (San Jose, California, 2016).

[25] Mohanty SP, Hughes DP, Salathé M (2016). Using deep learning for image-based plant disease detection. Front Plant Sci..

[26] Ferentinos, K. P. Deep learning models for plant disease detection and diagnosis. *Comput. Electron. Agric.* **145**, 311–318 (2018).

[27] Ramcharan A (2017). Deep learning for image-based cassava disease detection. Front Plant Sci..

[28] Ma J (2018). A recognition method for cucumber diseases using leaf symptom images based on deep convolutional neural network. Comput. Electron. Agric..

[29] Ubbens J, Cieslak M, Prusinkiewicz P, Stavness I (2018). The use of plant models in deep learning: an application to leaf counting in rosette plants. Plant Methods.

[30] Zhang Z (2012). Microsoft kinect sensor and its effect. IEEE Multimed..

[31] Xiong X (2017). Panicle - SEG: a robust image segmentation method for rice panicles in the field based on deep learning and superpixel optimization. Plant Methods.

[32] Vapnik VN (1999). An overview of statistical learning theory. IEEE Trans. Neural Netw..

[33] Smits, G. & Jordaan, E. M. Improved SVM regression using mixtures of kernels. In *Proc. 2002 International Joint Conference on Neural Networks*, 2785–2790 (Honolulu, Hawaii, 2002).

[34] Breiman L (2001). Random forests. Mach. Learn..

[35] Lin CH, Chen RT, Chan YK (2009). A smart content-based image retrieval system based on color and texture feature. Image Vis. Comput..

[36] Guo W, Rage UK, Ninomiya S (2013). Illumination invariant segmentation of vegetation for time series wheat images based on decision tree model. Comput. Electron. Agric.

[37] Donis-González IR, Guyer DE, Pease A (2016). Postharvest noninvasive classification of tough-fibrous asparagus using computed tomography images. Postharvest Biol. Technol..

[38] Xiong X (2017). A high‑throughput stereo‑imaging system for quantifying rape leaf traits during the seedling stage. Plant Methods.

[39] Hämmerle M, Höfle B (2016). Direct derivation of maize plant and crop height from low-cost time-of-flight camera measurements. Plant Methods.

[40] Andújar D, Ribeiro A, Fernández-quintanilla C, Dorado J (2016). Using depth cameras to extract structural parameters to assess the growth state and yield of cauliflower crops. Comput. Electron. Agric.

[41] Liu, M., Tuzel, O., Ramalingam, S. & Chellappa, R. Entropy rate superpixel segmentation. In Proc. 2011 IEEE Conference on Computer Vision and Pattern Recognition, 2097–2104 (Providence, Rhode Island, 2011).

[42] Yang S (2019). Method for measurement of vegetable seedlings height based on RGB-D camera. Trans. Chin. Soc. Agric. Machinery..

[43] Chen D (2014). Dissecting the phenotypic components of crop plant growthand drought responses based on high-throughput image analysis w open. Plant Cell Online.

[44] Golzarian MR, Frick RA, Rajendran K, Berger B, Lun DS (2011). Accurate inference of shoot biomass from high-throughput images of cereal plants. Plant Methods.

[45] Mortensen AK, Bender A, Whelan B, Barbour MM (2018). Segmentation of lettuce in coloured 3D point clouds for fresh weight estimation. Comput. Electron. Agric..

[46] Tudela JA, Hernández N, Pérez-Vicente A, Gil MI (2017). Postharvest biology and technology growing season climates affect quality of fresh-cut lettuce. Postharvest Biol. Technol..

[47] Lecun Y, Bengio Y, Hinton G (2015). Deep learning. Nature.

